# The association between pro-vegetarian dietary pattern and risk of colorectal cancer: a matched case-control study

**DOI:** 10.1186/s13104-023-06606-6

**Published:** 2023-11-09

**Authors:** Elham Tavassoli Nejad, Elham Moslemi, Fateme Souni, Marzieh Mahmoodi, Mohebat Vali, Mohammad Vatanpour, Mehran Nouri, Atena Ramezani, Zainab Shateri, Bahram Rashidkhani

**Affiliations:** 1 Department of Clinical Nutrition, School of Nutrition and Food Sciences, Isfahan University of Medical Sciences, Isfahan, Iran; 2 Student Research Committee, Faculty of Nutrition and Food Sciences, Tabriz University of Medical Sciences, Tabriz, Iran; 3 Department of Clinical Nutrition, School of Nutrition and Food Sciences, Shiraz University of Medical Sciences, Shiraz, Iran; 4 Nutrition Research Center, Shiraz University of Medical Sciences, Shiraz, Iran; 5 Student Research Committee, Shiraz University of Medical Sciences, Shiraz, Iran; 6 Department of Community Nutrition, School of Nutrition and Food Sciences, Shiraz University of Medical Sciences, Shiraz, Iran; 7 Diabetes Research Center, Mazandaran University of Medical Sciences, Sari, Iran; 8 Student Research Committee, Ahvaz Jundishapur University of Medical Sciences, Ahvaz, Iran; 9 Department of Community Nutrition, Faculty of Nutrition and Food Technology, National Nutrition and Food Technology Research Institute, Shahid Beheshti University of Medical Sciences, Tehran, Iran

**Keywords:** Pro-vegetarian dietary pattern, Colorectal Neoplasms, Colorectal cancer, Iranian

## Abstract

**Background:**

Few studies assess the link between plant-based diets and colorectal cancer (CRC) incidence. To our knowledge, no study has examined the association between pro-vegetarian dietary pattern (PDP) and CRC globally or among Iranians. Therefore, the objective of our matched case-control study was to evaluate the association between PDP and CRC in the Iranian population.

**Methods:**

The present research was a hospital-based case (n = 71)-control (n = 142) study conducted in the same hospitals in Tehran, Iran. This study used a reliable and valid semi-quantitative food frequency questionnaire to evaluate the participants’ dietary intake. According to the residual method, the selected plant and animal foods have been adjusted in the total energy intake to calculate the PDP index. Odds ratios (ORs) and 95% confidence intervals (CIs) adjusted for confounding variables were also expressed using logistic regression by SPSS software.

**Results:**

In the crude and adjusted models, we observed that the odds of CRC decreased significantly in the 3rd and last quartile of PDP compared to the 1st quartile (Q) (Crude model: Q_3_: OR = 0.36, 95% CI: 0.17 − 0.79, P-value = 0.011 and Q_4_: OR = 0.33, 95% CI: 0.14 − 0.79, P-value = 0.012 - Adjusted model: Q_3_: OR = 0.41, 95% CI: 0.18 − 0.94, P-value = 0.035 and Q_4_: OR = 0.35, 95% CI: 0.14 − 0.87, P-value = 0.025).

**Conclusions:**

Based on the results of the present case-control study in the Iranian population, it was concluded that PDP, which involves consuming vegetables, fruits, cereals, dairy products, and low meat consumption, reduces the odds of CRC. In conclusion, adherence to PDP is associated with a decreased odds of CRC.

## Introduction

Colorectal cancer (CRC) is an invasive and malignant disease in which the rectum and colon cells grow out of control [1]. In the world, CRC is the second and third most frequent cancer among women and men, respectively [2]. Even though there are more screening tests (mainly colonoscopies), CRC is still the third leading cause of cancer-related death worldwide [3]. CRC is also the third most common cause of death in Iran and has been on an upward trend over the past 25 years, particularly in younger populations [4].

Lifestyle factors, diet, age, and family history have been found to play a role in the pathogenesis of CRC [1]. Modifiable lifestyle factors contributing to the risk of CRC include consuming fewer fruits and vegetables, consuming more red meat, drinking more alcohol, smoking, consuming less calcium, being inactive, and having other diseases like obesity and type 2 diabetes [5]. To determine nutritional status, dietary patterns that include different food groups and their interactions between themselves and not individual foods should be used [6].

Nowadays, people are becoming more interested in the pro-vegetarian dietary pattern (PDP), which is based on consuming more plant- and less animal-based foods [7]. PDP and plant-based diets are known to be beneficial for non-communicable diseases such as cardiovascular disease [8], hypertension [9], type 2 diabetes [10], and cancer [11]. To assess adherence to a PDP distinct from complete vegetarianism, an overall PDP index was created for the first time, weighing both animal- and plant-derived foods [12]. Due to decreased meat consumption (especially red and processed meat) and increased high-fiber foods, PDP may be associated with a lower risk of CRC [13]. Additionally, plant-based diets are associated with lower body mass index (BMI) [14, 15], and there is strong evidence linking higher adiposity to an increased risk of CRC [16]. However, plant-based diets in Britain have not been associated with reduced CRC incidence [17]. Additionally, studies have shown that vegetarian diets are associated with lower rates of gastrointestinal cancers and overall cancer incidence but not with lower overall cancer death rates [18, 19]. On the other hand, the results of a previous cohort study showed that meat consumption was associated with an increased risk of colon cancer. In contrast, legume consumption was associated with a lower risk [15].

Examining dietary patterns rather than individual nutrients is recommended to clarify the relationship between diet and health [20]. Also, most available studies on cancers and dietary patterns have been collected from developed countries, with almost two-thirds of these studies being conducted in Europe or North America [21]. To our knowledge, no study has examined the association between PDP and CRC globally or among Iranians. Therefore, our matched case-control study aimed to investigate the association between PDP and CRC in the Iranian population.

## Methods

### Study population

The present research was a case-control study that was conducted in 19 CRC surgery departments of Imam Khomeini Hospital’s Cancer Organization and three general hospitals in Tehran, Iran (from September 2008 to January 2010). Participants who had the following conditions were included in the study as cases (convenience sampling): definite diagnosis of CRC for six months before the interview, ages between 40 and 75 years at the time of diagnosis, no history of inflammatory bowel disease and familial adenomatous polyposis and other cancers.

The subjects of the control group were randomly selected from the patients hospitalized in the same hospitals due to acute and non-neoplastic disorders such as joint and disc disorders, sprains, and fractures. Each case was age- and sex-matched with two control subjects.

Based on the sample size obtained from the previous study [22], 89 cases and 178 controls were included in the current study. Then, after excluding 54 participants due to unwillingness to cooperate, incomplete food frequency questionnaire (FFQ), and excessive intake of total energy (out of mean ± 3 standard deviations (SDs)), 71 cases and 142 controls were included in the final analysis. Some details of the present study have been previously published [23].

### Dietary intake

This study used a 168-item semi-quantitative FFQ to evaluate the participants’ dietary intake, the reliability and validity of which had been previously assessed [24]. A trained dietitian completed the questionnaire through a face-to-face interview. Participants ' dietary intake in the previous year was estimated using a valid food album and standard measurement tools [25]. Finally, after converting participants’ daily dietary intake into grams, we used Nutritionist IV (N IV) to estimate energy and nutrient intake [26].

### PDP

According to the residual method, the selected plant and animal foods were adjusted in the total energy intake to calculate the PDP index. Potatoes, olive oil, nuts, cereals, legumes, vegetables, and fruits were considered as plant sources, and meat products, animal fat, eggs, fish, and dairy products were categorized as animal sources. After that, all plant and animal components were converted into a quantile score, and then a reverse score was considered for the quantiles of animal components. Finally, the value of the reverse quantile of animal sources and direct quantile of plant sources were calculated. After determining the values of these plant and animal foods, the final PDP score (between 12 and 60) was calculated [7].

### Assessment of covariates

A general information checklist was used to collect the participants’ general information, including family history of CRC, history of drug use, and socio-demographic characteristics. Smoking status was classified as never, former, or current. Those who had never smoked or had smoked less than 100 cigarettes in their lifetime were considered never smokers. Former smokers were considered those who had quit smoking at the time of the interview and had smoked at least 100 cigarettes in their lifetime. Current smokers were defined as those who currently smoke and have smoked at least 100 cigarettes in their lifetime. Anthropometric parameters, including weight, height, and waist-to-hip ratio (WHR), were evaluated through standard methods. Also, each participant’s BMI was calculated. The level of physical activity was determined using an International Physical Activity Questionnaire (IPAQ), previously validated in the Iranian population [27].

### Statistical analysis

Statistical analyses were performed using a statistical software package (SPSS, version 26). Also, we used R software for all the depicted figures. The dietary intake and basic characteristics of the participants were expressed as mean ± SD (for data with a normal distribution) or median (interquartile range (IQR) for data with a non-normal distribution) and number (percentage) for quantitative and qualitative data, respectively. Independent samples T-test or Mann-Whitney and chi-square were used to compare continuous and categorical variables between two groups, respectively. Analysis of variance (ANOVA) test was used to assess the PDP component intakes across the quartile of this index. Odds ratios (ORs) and 95% confidence intervals (CIs) adjusted for confounding variables were also expressed using logistic regression. The significance level for all findings was considered as a p-value less than 0.05.

## Results

According to Table 1, the two case and control groups significantly differed in the history of CRC, WHR, PDP score, fiber intake, and taking aspirin, acetaminophen, and mineral supplements (P < 0.05).

**Table 1:** Basic characteristics of the study participants in the case and control groups

Variables	Cases (71)	Controls (142)	P-value
Gender ^1^			1
Male	35 (49.3)	70 (49.3)	
Female	36 (50.7)	72 (50.7)	
Common ways of cooking meat ^1^			0.282
Fried	20 (28.2)	28 (19.7)	
Fried / Boiling	35 (49.3)	71 (50.0)	
Smoking / Grilling	16 (22.5)	43 (30.3)	
Common ways of preparing vegetables ^1^			0.083
Raw / Fresh	29 (40.8)	78 (54.9)	
Boiled	8 (11.3)	18 (12.7)	
Fried, Fried / Frozen	34 (47.9)	46 (32.4)	
Family history of CRC in the first-degree relatives ^1^			**0.017**
Yes	7 (9.9)	3 (2.1)	
No	64 (90.1)	139 (97.9)	
Education ^1^			0.147
No formal education	28 (39.3)	36 (25.4)	
Elementary	22 (31.0)	45 (31.6)	
Junior/ High school	7 (9.9)	19 (13.4)	
Diploma/College/University	14 (19.7)	42 (29.6)	
Smoking ^1^			0.164
Never	57 (80.2)	101 (70.1)	
Former	8 (11.3)	15 (10.6)	
Current	6 (8.5)	26 (18.3)	
Age (year) ^2^	58.2 ± 10.4	57.7 ± 10.4	0.746
BMI (kg/m^2^) ^2^	27.6 ± 4.2	26.6 ± 4.2	0.362
WHR ^2^	0.97 ± 0.08	0.94 ± 0.06	**0.002**
Income (dollar/month) ^3^	393.0 (253.0)	402.0 (302.0)	0.206
Physical activity (MET-h/day) ^2^	36.8 ± 3.6	36.7 ± 4.8	0.932
PDP score ^2^	34.9 ± 4.7	36.3 ± 4.6	**0.043**
Energy (kcal/day) ^2^	2262.3 ± 450.1	2255.2 ± 341.2	0.908
Fiber (g/day) ^2^	18.9 ± 2.3	20.4 ± 3.1	**<0.001**
Ibuprofen ^1^			0.059
Yes	5 (7.0)	22 (15.5)	
No	66 (93.0)	120 (84.5)	
Aspirin ^1^			**0.016**
Yes	1 (1.4)	14 (9.9)	
No	70 (98.6)	128 (90.1)	
Acetaminophen ^1^			**0.004**
Yes	4 (5.6)	28 (19.7)	
No	67 (94.4)	114 (80.3)	
Baby aspirin ^1^			0.106
Yes	15 (21.1)	19 (13.4)	
No	56 (78.9)	123 (86.6)	
Mineral supplement use ^1^			**0.015**
Yes	8 (11.3)	35 (24.6)	
No	63 (88.7)	107 (75.4)	

BMI: body mass index, WHR: waist-to-hip ratio, MET: metabolic equivalent of task, PDP: pro-vegetarian dietary pattern, CRC: colorectal cancer, g: gram, h: hour

Significant values are shown in bold.

Using chi-square test for categorical and Mann-Whitney or independent samples T-test for continuous variables

^1^Values are number (percent).

^2^Values are mean ± SD.

^3^Values are median (IQR).

**Fig. 1:**
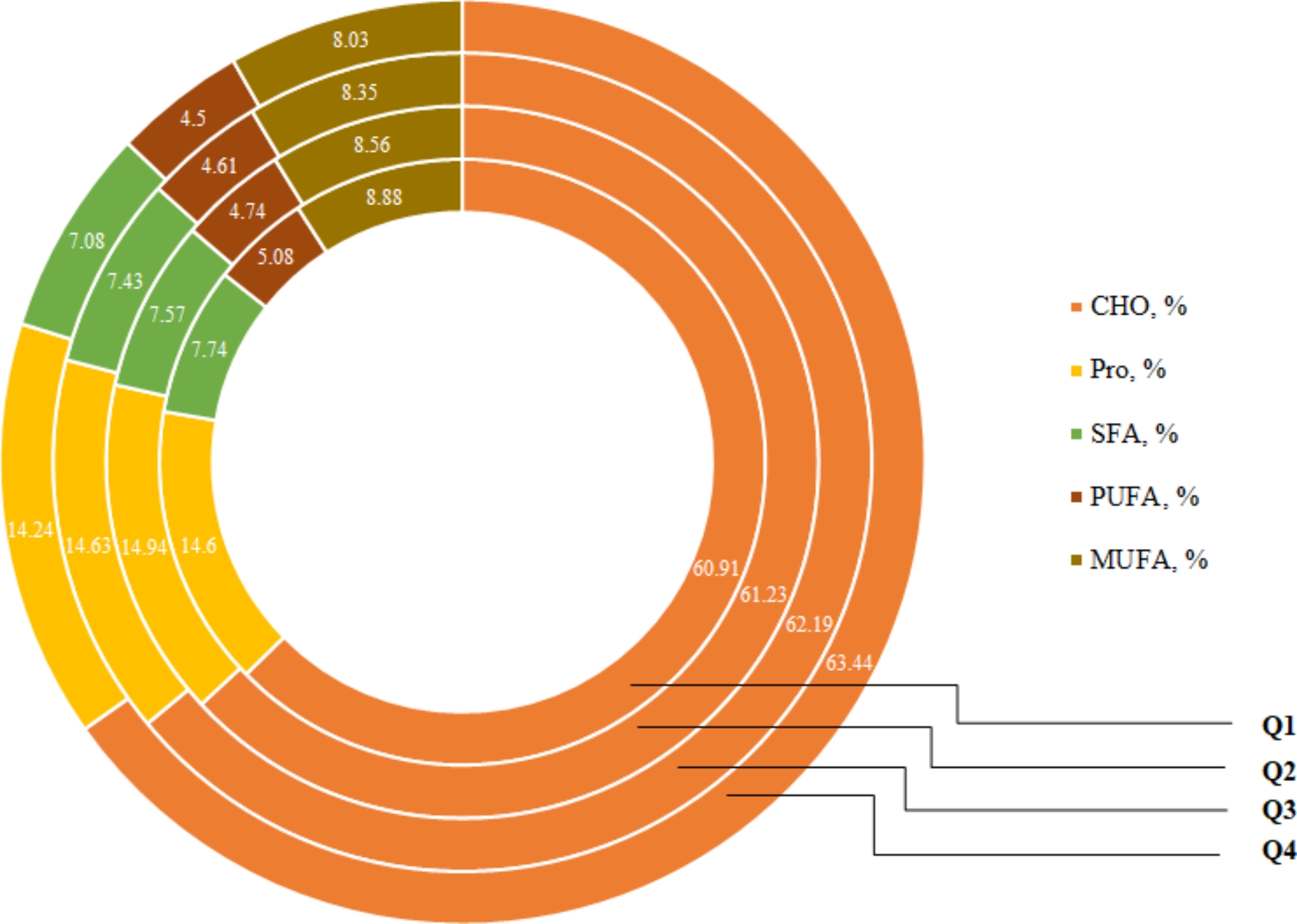
Macronutrient intakes based on the PDP quartiles. PDP: pro-vegetarian dietary pattern, Q: quartile, CHO: carbohydrate, Pro: protein, SFA: saturated fatty acid, PUFA: polyunsaturated fatty acid, MUFA: monounsaturated fatty acid

According to Fig. 1, carbohydrate intake was significantly higher and polyunsaturated fatty acids (PUFAs) lower in the last quartile of the PDP score compared to the first one (P = 0.048 and P = 0.045, respectively).

The consumption of plant and animal sources based on PDP quartiles is presented in Table 2. Fruits (P<0.001), vegetables (P<0.001), nuts (P<0.001), cereals (P = 0.009), legumes (P = 0.009), olive oil (P<0.001), and potato intake (P = 0.006) were significantly higher in the last quartiles of PDP. Compared to the first quartile of the PDP score, animal fat consumption (P<0.001) was significantly lower in the last quartile.

**Table 2:** Intake of PDP components based on the PDP quartiles

Variables	Q1 (n = 51)	Q2 (n = 55)	Q3 (n = 69)	Q4 (n = 49)	P-value
Fruits (g/day)	199.0 (117.4)	233.2 (135.0)	317.0 (269.9)	286.5 (222.1)	**<0.001**
Vegetables (g/day)	109.4 (72.4)	136.6 (74.0)	209.8 (109.0)	207.3 (109.0)	**<0.001**
Nuts (g/day)	1.6 (2.0)	2.6 (4.1)	3.9 (7.9)	5.9 (7.4)	**<0.001**
Cereals (g/day)	357.5 (195.6)	355.4 (206.9)	388.8 (233.6)	436.3 (270.2)	**0.009**
Legumes (g/day)	19.4 (21.9)	21.4 (22.1)	21.8 (26.6)	31.4 (26.1)	**0.009**
Olive Oil (g/day)	0.0 (0.8)	0.2 (2.1)	1.0 (4.5)	1.4 (3.3)	**<0.001**
Potatoes (g/day)	10.7 (9.6)	15.2 (11.8)	17.4 (16.5)	17.4 (21.3)	**0.006**
Animal Fat (g/day)	8.2 (17.8)	6.0 (9.1)	3.9 (7.7)	1.9 (4.4)	**<0.001**
Eggs (g/day)	20.1 (14.1)	19.6 (14.1)	18.9 (7.0)	13.0 (10.2)	0.069
Fish (g/day)	6.3 (8.6)	5.2 (8.7)	5.8 (11.6)	6.3 (8.1)	0.499
Dairy Products (g/day)	250.0 (213.2)	164.1 (289.5)	248.9 (209.8)	184.3 (315.4)	0.693
Meats and Processed Meats (g/day)	33.6 (24.3)	28.8 (21.5)	36.0 (33.3)	29.2 (19.8)	0.124

PDP: pro-vegetarian dietary pattern, Q: quartile, g: gram

Using Kruskal-Wallis U test

^3^Values are median (IQR).

The association between PDP score and CRC odds is shown in Table 3. In the crude and adjusted models, we observed that the odds of CRC decreased significantly in the 3rd and last quartile of PDP compared to the 1st quartile (Q) (Crude model: Q_3_: OR = 0.36, 95% CI: 0.17 − 0.79, P-value = 0.011 and Q_4_: OR = 0.33, 95% CI: 0.14 − 0.79, P-value = 0.012 - Adjusted model: Q_3_: OR = 0.41, 95% CI: 0.18 − 0.94, P-value = 0.035 and Q_4_: OR = 0.35, 95% CI: 0.14 − 0.87, P-value = 0.025).

**Table 3:** Association between PDP and colorectal cancer

Quartiles of PDP	Case/Control	Model 1			Model 2		
		OR	95% CI	P-value	OR	95% CI	P-value
Q_1_ (≤ 32)	25/26	1.00	Ref.		1.00	Ref.	
Q_2_ (33–35)	16/28	0.59	0.26–1.35	0.216	0.72	0.29–1.77	0.485
Q_3_ (36–39)	18/51	**0.36**	**0.17–0.79**	**0.011**	**0.41**	**0.18–0.94**	**0.035**
Q_4_ (≥ 40)	12/37	**0.33**	**0.14–0.79**	**0.012**	**0.35**	**0.14–0.87**	**0.025**
P_trend_		**0.004**			**0.010**		

PDP: pro-vegetarian dietary pattern, Q: quartile, CI: confidence interval, OR: odds ratio

Significant values are shown in bold.

These values are odds ratio (95% CIs).

Obtained from logistic regression

Model 1: crude model

Model 2: adjusted for energy intake, history of CRC, common ways of preparing vegetables, common ways of cooking meats, physical activity, BMI, and smoking

## Discussion

The present matched case-control study examined PDP as a plant-based dietary pattern to determine whether it is associated with CRC odds in the Iranian population. We observed that a PDP characterized by frequent fruit, vegetable, cereal, and dairy product consumption was associated with a decreased odds of CRC for both men and women.

Previous studies showed the beneficial effects of various plant-based diets, such as a vegetarian diet [28, 29]. However, due to the elimination of animal products in this dietary pattern, sufficient amounts of certain nutrients such as iron, vitamin B_12_, and long-chain fatty acids (eicosapentaenoic acid and docosahexaenoic acid) are not provided [30]. Some studies have shown a link between a decrease in the intake of the mentioned nutrients and a higher incidence of cancer [31, 32]. Therefore, a PDP diet with a certain amount of meat, fish, egg, and dairy is one of the best alternative diets that provide enough macro- and micronutrients [33].

There has been no research on the relationship between PDP and CRC, so we investigated the relationship between various types of plant-based diets and CRC, and the results of previous studies have been conflicting in this regard [34, 35]. Like our findings, Wirfält et al., in a prospective cohort study of people aged 50 to 71 years (n = 492,306), found that consuming vegetables and fruits reduced CRC risk by 15% [35]. In a case (n = 506)-control (n = 673) study conducted on the Canadian population, Chen et al. identified a plant-based dietary pattern associated with a lower odds of CRC (OR = 0.55) [36]. In the present study, a greater odds reduction was observed between PDP and CRC (OR = 0.35). Additionally, low-meat eaters had a 9% lower CRC risk than regular meat eaters in the prospective analysis of the United Kingdom Biobank study [37]. Findings from a multiethnic cohort study on men (n = 79,952) and women (n = 93,475) showed that a plant-based diet was significantly associated with a lower CRC risk only in men. Because men are more likely to suffer from CRC than women, and these two genders have different dietary habits [38]. Previous studies have shown that whole grains, cereals, or vegetables are inversely associated with cancer risk from the cecum to the rectum, indicating a close relationship between diet and CRC [39]. In line with our results, studies have shown that a healthy plant-based diet was inversely correlated with CRC incidence [40, 41]. Also, a systematic review and meta-analysis of observational studies showed a significant reduction in total cancer incidence among vegans and vegetarians as plant-based diets [42].

In contrast to our results, a case-control study on the Malaysian population (n = 264) showed no significant relationship between a plant-based diet and the risk of CRC [43]. Also, a cohort study found no association between vegetarianism and CRC risk [34]. The definitions of vegetarian and other plant-based diets and the time participants followed their respective diets also varied between studies. A clear conclusion cannot be drawn from the small number of cases of CRC in some dietary groups, such as PDP. However, a Northern German prospective cohort study of 1,404 CRC survivors also showed that compared to the 1st quartile after age and sex adjustment, those in the last quartile of a plant-based diet had lower mortality in survivors of CRC during seven years of follow-up [44].

The benefits of a plant-based diet, particularly PDP, on CRC risk can be attributed to several mechanisms. Many healthy plant foods in the PDP, such as fruits, vegetables, and cereals, contain dietary fiber, polyphenols, carotenoids, lignans, and vitamins E and C [45, 46]. Due to their antioxidant and anti-inflammatory properties, these functional nutritional components can cause tumor cells to undergo apoptosis and reduce CRC incidence [47]. Also, the fiber in the PDP may mitigate the risk of CRC by shortening the time it takes to move through the body and producing short-chain fatty acids [40, 48]. Short-chain fatty acids prevent the onset of infection and cancer by affecting the immune system and gene expression [49].

Moreover, red meat can cause cancer because it contains protein, fat, iron, or heat-induced mutagenic substances [50]. In processed meat, in addition to the mentioned items, salt and nitrite are added during processing [51]. Also, there is evidence that red meat and processed meats produce genotoxic free radicals and lipid peroxidation, which are associated with altered colonic flora and increased risk of CRC [52]. In addition, excessive fat intake can cause weight gain and insulin resistance [50]. As a result, blood sugar, insulin, and insulin-like growth factor 1 (IGF-1) increase, which causes the proliferation of precancerous cells and stops apoptosis [53].

Some studies show the effect of smoking and being overweight on cancer risk [54, 55]. In our research, all participants were overweight (differences between the BMI of the case and control were not significant), and the impact of BMI and smoking were adjusted in the second model. The association between PDP and CRC was still significant despite considering smoking and BMI in the adjusted model. Therefore, further research is needed to understand the mechanisms underlying our findings.

### Strengths and limitations

To our knowledge, this is the first study to address the association between PDP and CRC in an Iranian population and provides up-to-date information to inform public health action for primary prevention. Also, instead of using a single nutrient/food approach as dietary intake and nutritional status indicators, food group analysis was used to investigate dietary patterns.

Selection bias and recall bias were two limitations of case-control studies, so in the present study, we matched both groups based on age and gender to control bias. Although the present study had a relatively small sample size, we selected twice as many people from the control group as the case group. Recall bias was also reduced by a validated FFQ and trained interviewers who were unaware of the study’s hypotheses.

In this study, assessing the long-term effects of risk factors on CRC rates was impossible. A cohort or longitudinal study is a better way to determine the association of lifestyle factors with long-term diseases such as cancer. Also, one of the main limitations of the current study was its hospital-based design.

## Conclusions

Based on the results of the present case-control study in the Iranian population, it was concluded that PDP, which includes the consumption of vegetables, fruits, cereals, and dairy products and low meat consumption, reduces the odds of CRC. In conclusion, adherence to PDP is associated with a reduced odds of CRC.

## Data Availability

The datasets used and/or analyzed during the current study are available from the corresponding author on reasonable request.
